# Pan-cancer Analysis Identifies AIMP2 as a Potential Biomarker for Breast Cancer

**DOI:** 10.2174/0113892029255941231014142050

**Published:** 2023-12-20

**Authors:** Jie Qiu, Tao Zhou, Danhong Wang, Weimin Hong, Da Qian, Xuli Meng, Xiaozhen Liu

**Affiliations:** 1 Department of Breast and Thyroid Surgery, Shaoxing People’s Hospital, Shaoxing 312000, Zhejiang, China;; 2 General Surgery, Cancer Center, Department of Breast Surgery, Zhejiang Provincial People’s Hospital, Hangzhou Medical College, Hangzhou 310000, Zhejiang, China;; 3 College of Pharmacy, Zhejiang University of Technology, Hangzhou 310014, Zhejiang, China;; 4 Department of Burn and Plastic Surgery-Hand Surgery, Changshu Hospital Affiliated to Soochow University, Changshu No.1 People’s Hospital, Changshu 215500, Jiangsu Province, China

**Keywords:** AIMP2, biomarker, breast cancer, immune microenvironment, pan-cancer, multifunctional protein 2

## Abstract

**Introduction:**

Aminoacyl tRNA synthetase complex interacting with multifunctional protein 2 (AIMP2) is a significant regulator of cell proliferation and apoptosis. Despite its abnormal expression in various tumor types, the specific functions and effects of AIMP2 on tumor immune cell infiltration, proliferation, and migration remain unclear.

**Materials and Methods:**

To assess AIMP2's role in tumor immunity, we conducted a pan-cancer multi-database analysis using the Cancer Genome Atlas (TCGA), Genotype-Tissue Expression (GTEx), and Cancer Cell Lines Encyclopedia (CCLE) datasets, examining expression levels, prognosis, tumor progression, and immune microenvironment. Additionally, we investigated AIMP2's impact on breast cancer (BRCA) proliferation and migration using cell counting kit 8 (CCK-8) assay, transwell assays, and western blot analysis.

**Results:**

Our findings revealed that AIMP2 was overexpressed in 24 tumor tissue types compared to normal tissue and was associated with four tumor stages. Survival analysis indicated that AIMP2 expression was strongly correlated with overall survival (OS) in certain cancer patients, with high AIMP2 expression linked to poorer prognosis in five cancer types.

**Conclusion:**

Finally, siRNA-mediated AIMP2 knockdown inhibited BRCA cell proliferation and migration *in vitro*. In conclusion, our pan-cancer analysis suggests that AIMP2 may play a crucial role in tumor immunity and could serve as a potential prognostic marker, particularly in BRCA.

## 1 INTRODUCTION

Aminoacyl tRNA synthetase complex interacting multifunctional protein 2 (AIMP2), also known as JTV1, is a multifunctional protein that forms a macromolecular complex with human aminoacyl tRNA synthetase. This complex comprises three non-enzymatic proteins: p43, p38, and p18, with p38 protein identified as AIMP2 [1]. AIMP2 is essential for the assembly and stability of the aminoacyl tRNA synthetase complex [2].

In addition to its importance in efficient protein synthesis, AIMP2 has been found to have other physiological roles [3, 4]. For instance, after DNA damage, AIMP2 is released from the ARS complex, phosphorylated through a JNK2-dependent pathway, and translocated into the nucleus, where it is proposed to bind and sequester p53 from Mdm2-dependent ubiquitination [5]. AIMP2 has also been demonstrated to be a substrate of E3 ligase Parkin [6]. Accumulation of AIMP2 due to Parkin mutation has been hypothesized to contribute to dopaminergic cell death observed in Parkinson's patients [7]. Moreover, AIMP2 enhances tumor necrosis factor-α-induced apoptotic signaling and exhibits antiproliferative activities in TGF-β and Wnt pathways through distinct mechanisms [8-10]. Therefore, we hypothesize that AIMP2 may play a critical role in cancer initiation and progression. However, there is a scarcity of research on AIMP2 in oncology. Existing studies suggest that AIMP2 may function as a multifaceted tumor suppressor [9, 11].

In this study, we investigated the expression of AIMP2 and its relationship with prognosis, Tumor Mutation Burden (TMB), and microsatellite instability (MSI) across 33 cancer types. Additionally, we explored the correlation between AIMP2 and the immune microenvironment, immune-related antigens, and immune checkpoint genes. Our findings indicated that AIMP2 was more highly expressed in tumor tissue than in normal tissue and was associated with various tumor stages. Survival analysis revealed a strong association between AIMP2 expression and overall survival (OS) in certain cancer patients, where high AIMP2 expression correlated with worse prognosis in five types of cancer. Furthermore, we confirmed that the expression level of AIMP2 was associated with tumor immune infiltration and the tumor microenvironment, particularly in breast cancer (BRCA). Lastly, siRNA-mediated knockdown of AIMP2 inhibited the proliferation and migration of breast cancer cells *in vitro*. In conclusion, our pan-cancer analysis identified differential expression of AIMP2, suggesting its potential importance in tumor immunity and its promise as a potential prognostic marker, especially for BRCA.

## 2 MATERIALS AND METHODS

### 2.1 TCGA Data Acquisition and Variance Analysis

The Cancer Genome Atlas (TCGA) database (https://portal.gdc.cancer.gov/) is a comprehensive resource containing gene expression data, copy number variations, and single nucleotide polymorphisms (SNPs), among other data. Raw mRNA expression data and SNP data from 33 pan-cancer tumor types were downloaded for subsequent analyses (Supplementary material **S1**) [12]. Gene expression data from different tissues were obtained from the Genotype-Tissue Expression (GTEx) database (https://commonfund.nih.gov/GTEx), merged with TCGA data, and adjusted to calculate gene expression differences across various cancers (Supplementary material **S2**) [13]. Data for each tumor cell line were acquired from the Cancer Cell Line Encyclopedia (CCLE) database (https://portals.broadinstitute.org/ccle/) and analyzed for gene expression levels according to tissue origin (Supplementary material **S3**) [14]. Furthermore, the correlation between expression and tumor stage was examined.

### 2.2 Prognostic Correlation Analysis

OS data of TCGA patients were downloaded from the Xena database (http://xena.ucsc.edu/) to investigate the relationship between gene expression and patient prognosis [15]. Survival analysis for each cancer type (*p* < 0.05) was conducted using the Kaplan-Meier method, and the “survival” and “survminer” packages were utilized to assess the survival analysis. Moreover, Cox analysis was performed with the “survival” and “forestplot” packages to explore the association between gene expression and survival.

### 2.3 Analysis of Immune Cell Infiltration

The CIBERSORT algorithm was employed to analyze RNA-seq data from 33 cancer patients in different subgroups to estimate the relative proportions of immune-infiltrating cells, as well as to examine the correlation between gene expression and immune cell content. Additionally, potential associations between gene expression and immune regulators (*e.g*., chemokines, immunosuppressants, immune stimulators, and major histocompatibility complex (MHC) molecules) were investigated using the Tumor-Immune System Interactions Database (TISIDB) website (cis.hku.hk/TISIDB/) [16].

### 2.4 Drug Sensitivity Analysis

The CellMiner database (https://discover.nci.nih.gov/cellminer/home.do) is based on the National Cancer Institute's (NCI) Center for Cancer Research's 60 cancer cell lines (NCI-60) [17, 18]. The NCI-60 cell line panel is currently the most widely used cancer cell sample population for anti-cancer drug testing [19]. In this study, NCI-60 drug sensitivity data and RNA-seq gene expression data were downloaded, and the relationship between genes and common anti-tumor drug sensitivity was explored through correlation analysis. A *p*-value of less than 0.05 was considered statistically significant.

### 2.5 GSVA Enrichment Analysis

Gene set variation analysis (GSVA) is an unsupervised, nonparametric method for assessing gene set enrichment within transcriptomes. GSVA comprehensively scores gene sets of interest, converting gene-level alterations into pathway-level changes, thus enabling the evaluation of the biological function of samples. In this study, gene sets were obtained from the Molecular Signatures Database, and the GSVA algorithm was employed to thoroughly score each gene set, assessing potential biological function changes in different samples [20].

### 2.6 GSEA Enrichment Analysis

Gene set enrichment analysis (GSEA) involves ranking genes based on their degree of differential expression between two sample types using a predefined set of genes. GSEA then tests whether the predefined gene set is enriched at the top or bottom of the ranked list. The “clusterprofiler” and “enrichplot” packages were utilized for GSEA analysis in this study [21]. By comparing differences in signaling pathways between high and low gene expression groups, potential molecular mechanisms underlying the prognostic differences among patients with 33 tumors were investigated.

### 2.7 Analysis of TMB and MSI Data

TMB is defined as the total number of somatic genetic coding errors, base substitutions, insertions, or deletions detected per megabase [22]. In this study, TMB was determined by dividing the number of nonsynonymous mutation sites by the total length of the protein-coding region, calculating the variant frequency and the number of variants per exon length for each tumor sample. The microsatellite instability (MSI) value for each TCGA patient was obtained from a previously published study [23].

### 2.8 Nomogram Model Construction

Nomograms are based on regression analysis and utilize linear segments with scales drawn on the same plane according to a specific proportion to represent the interactions between variables in a prediction model. By constructing a multifactorial regression model, scores are assigned to each value level of each influencing factor based on their contribution to the outcome variable (*i.e*., the regression coefficient's magnitude). The sum of these scores generates a total score, which is then used to calculate the predicted value.

### 2.9 Weighted Gene Co-expression Network Analysis (WGCNA)

To identify co-expressed gene modules and investigate their relationships with AIMP2 and core genes within the network, we constructed a weighted gene co-expression network. The WGCNA R package facilitated the construction of a co-expression network for all genes in the breast cancer dataset, and the top 5,000 genes with the highest variance were selected for further analysis [24]. A weighted adjacency matrix was transformed into a topological overlap matrix (TOM) to estimate network connectivity, and hierarchical clustering was employed to generate a clustering tree structure based on the TOM matrix. The distinct branches of the clustering tree represented different gene modules, with varying colors signifying unique modules. Genes were classified according to their expression patterns based on weighted correlation coefficients, grouped into modules based on similar patterns, and divided into multiple modules according to gene expression patterns.

### 2.10 *In Vitro* Validation

To examine the biological function of AIMP2 in breast cancer progression, we conducted immunohistochemistry and silenced AIMP2 expression in human breast cancer cell lines BT-549 and MDA-MB-231. AIMP2 siRNA was obtained from GenePharma. The cell counting kit-8 (CCK8) assay (Beyotime, Shanghai, China) evaluated cell viability following AIMP2 silencing. The transwell assay (champer purchase from Corning, NY, USA) verified the reduced invasion capacity of tumor cells after AIMP2 knockdown, and the wound healing assay confirmed the decreased migration ability of BRCA. Western blot analysis validated AIMP2 (10424-1-AP, 1:2000, Proteintech) protein level knockdown, and real-time quantitative PCR detection (Roche Light Cycler 480 QPCR instrumentation, Germany) confirmed AIMP2 mRNA knockdown efficiency. Additionally, immunohistochemical analysis of human normal and tumor tissues assessed AIMP2 expression. AIMP2 siRNA transfection into cells was performed using Lipofectamine RNAi MAX (Invitrogen, Carlsbad, CA), according to the manufacturer's instructions.

The following siRNA sequences were used:

AIMP2 siRNA-1: 5’- CACGACUUUAACCACCAAUTT-3’

AIMP2 siRNA-2: 5’-GUUGAAAGCUGCAGUUGAUTT-3’

AIMP2 siRNA-3: 5’-GCCAGAAGCAUAAUGCUGUTT-3’

The following primers were used:

AIMP2-forward primer: GAGGCAGGAGAATCGCTTGAACC

AIMP2-reverse primer: TCTAACCGACTCCGCCACTTCC

GAPDH-forward primer: AGAAAAACCTGCCAAATATGA TGAC

GAPDH-reverse primer: TGGGTGTCGCTGTTGAAGTC

### 2.11 Statistical Analysis

All statistical analyses were executed in R Studio (R version 4.0.2) software. Univariate survival analysis calculated hazard ratios (HRs) and 95% confidence intervals. Kaplan-Meier survival curve analysis examined patient survival based on high or low gene expression levels, with *p* < 0.05 deemed statistically significant. *In vitro* experiments, western blot bands, and transwell assay data were analyzed using ImageJ software. Statistical significance was determined with GraphPad Prism 9. Data are presented as mean ± SD. Student's t-test and one-way ANOVA were employed for data statistical analyses, and a *p*-value < 0.05 was considered statistically significant.

## 3 RESULTS

### 3.1 Pan-cancer Expression Analysis and Prognostic Value of the AIMP2 Gene

**Fig. (1):**
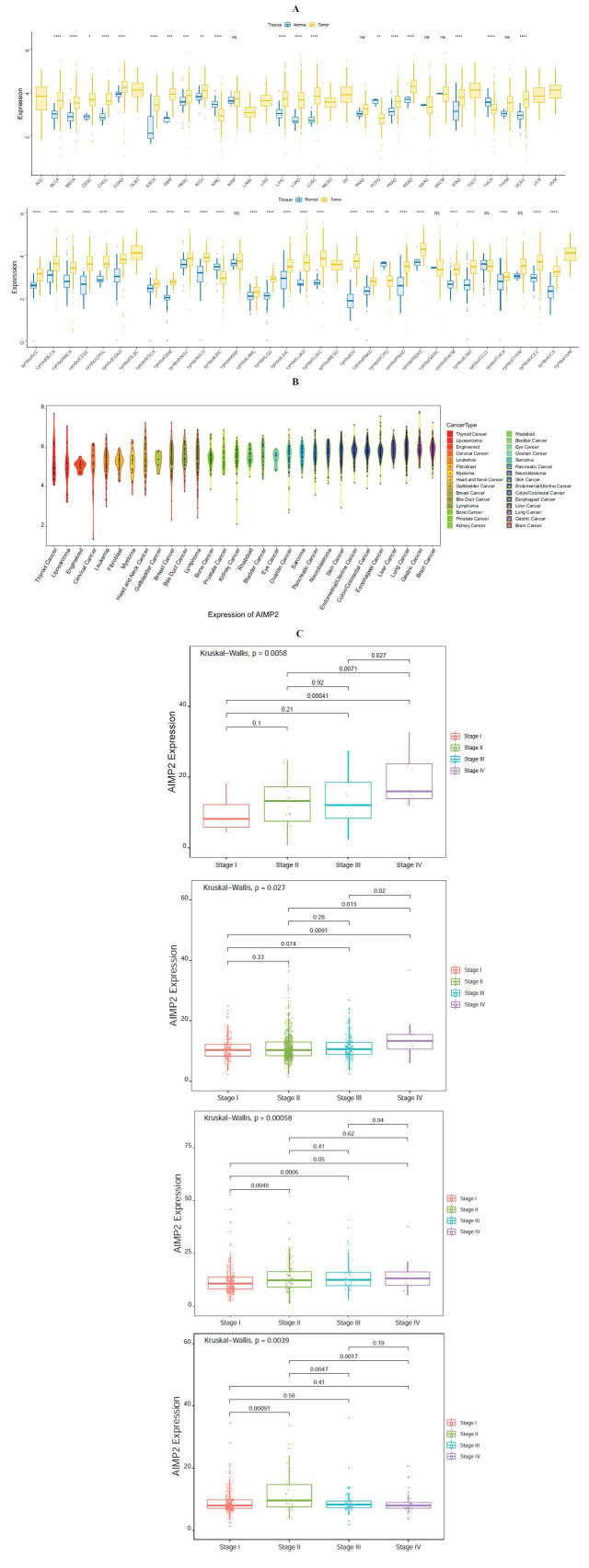
Pan-cancer analysis of AIMP2 expression. (**A**) Differential expression of AIMP2 between tumor and normal tissues in 33 human cancers from TCGA database (above) and GTEx database (below). (**B**) AIMP2 expression in different tumor cell lines. (**C**) AIMP2 expression significantly differs in various stages of ACC, BRCA, LUAD, and THCA (*p* < 0.05). (ACC: adrenocortical carcinoma; BRCA: breast invasive carcinoma; LUAD: lung adenocarcinoma; THCA: thyroid carcinoma).

**Fig. (2):**
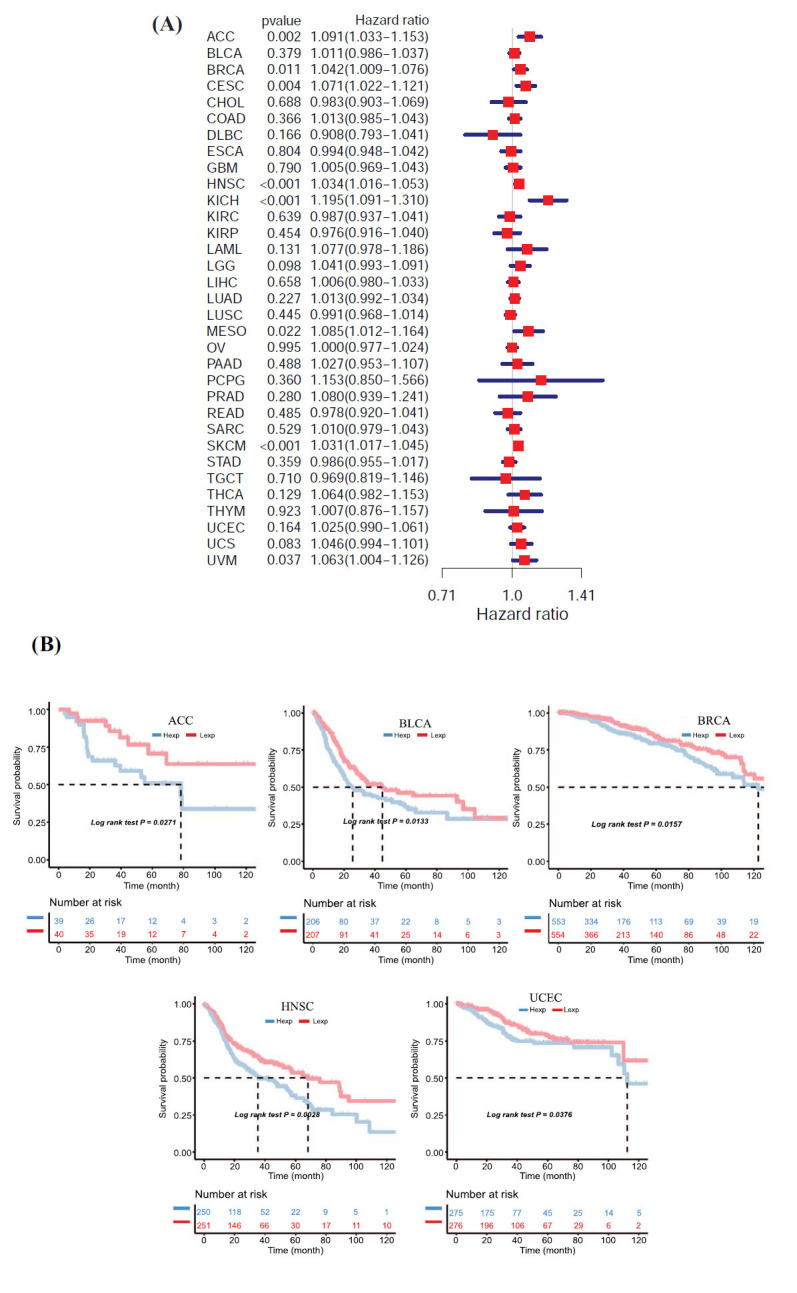
AIMP2 expression correlates with overall survival time (OS). (**A**) Forest plots showing correlations between OS and AIMP2 expression across 33 cancer types. (**B**) Kaplan-Meier analyses of the association between AIMP2 expression and OS in ACC, BLCA, BRCA, HNSC, and UCEC, respectively. (Hexp: high expression; Lexp: low expression).

We analyzed the expression of AIMP2 in 33 human cancers using TCGA and GTEx datasets, respectively (Fig. **1A**). In the majority of cancer tissues, AIMP2 expression levels were higher than those in normal tissues. AIMP2 expression in various tumor cell lines from the CCLE expression profile is depicted in Fig. **1B**. The levels of expression decreased sequentially from left to right. Furthermore, we observed an association between AIMP2 expression and multiple tumor stages, including ACC, BRCA, LUAD, and THCA, respectively (Fig. **1C**). To assess the relationship between AIMP2 expression and cancer patient prognosis, we found that AIMP2 expression strongly correlated with OS in eight cancer types: ACC, BRCA, CESC, HNSC, KICH, MESO, SKCM, and UVM tumors (Fig. **2A**). Moreover, Kaplan-Meier survival analysis results indicated that high AIMP2 expression correlated with poorer OS in five cancer types: ACC, BLCA, BRCA, HNSC, and UCEC (Fig. **2B**).

### 3.2 Pan-cancer Expression and Immune Infiltration

**Fig. (3):**
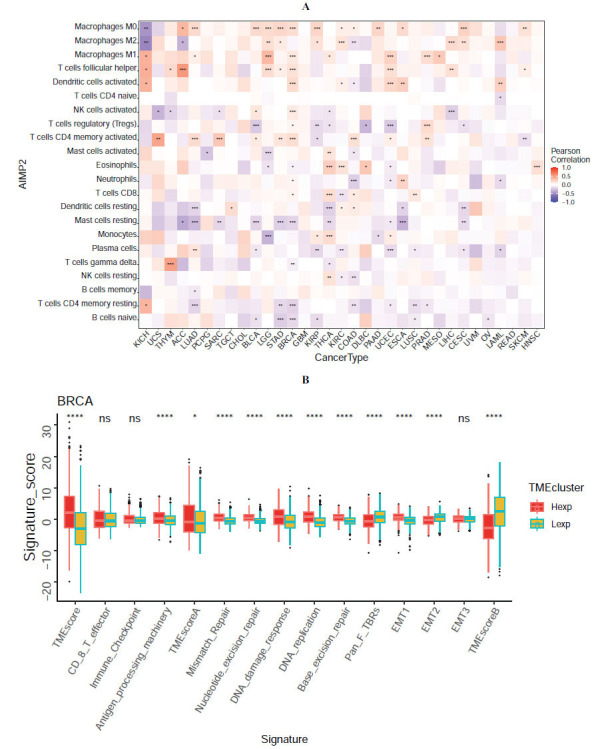
Pan-cancer analysis of the relationship between AIMP2 expression and immune cell infiltration. (**A**) Correlation between AIMP2 expression and immune cells in 33 cancer types. (**B**) Correlation analysis between tumor microenvironment and breast cancer. (* *p* < 0.05; ** *p* < 0.01; **** *p* < 0.0001).

The tumor microenvironment (TME) is a complex milieu consisting of tumor-associated fibroblasts, immune cells, extracellular matrix, diverse growth factors, inflammatory factors, unique physicochemical properties, and cancer cells. The TME substantially influences tumor diagnosis, survival, and clinical sensitivity. Our findings revealed a strong association between AIMP2 expression and immune infiltration, with 14 cancers significantly correlated with M0 macrophages, 10 cancers significantly correlated with M2 macrophages, and 8 cancers significantly correlated with M1 macrophages (Fig. **3A**). We conducted further TME analysis on BRCA and found significant correlations between TMEscore, Antigen_processing_machinery, TME scoreA, Mismatch_Repair, Nucleotide_excision_repair, DNA damage response, DNA_replication, Base_excision_ repair, Pan_F_TBRs, EMT1, EMT2, and TMEscoreB scores and breast cancer (Fig. **3B**).

### 3.3 Pan-cancer Expression and Key Regulatory Genes

**Fig. (4):**
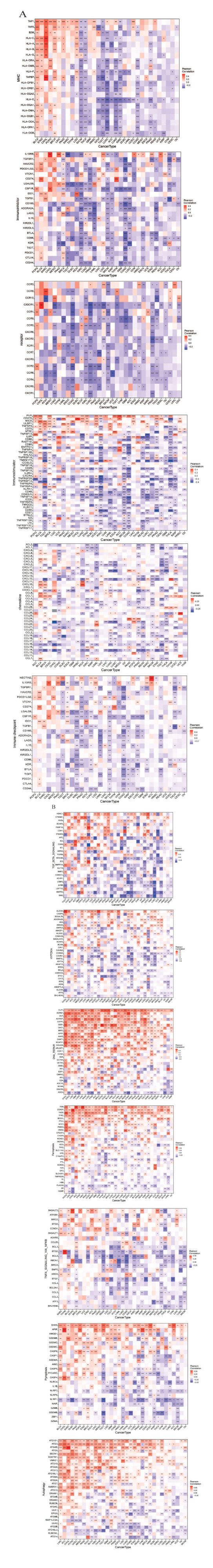
Analysis of AIMP2 expression and its correlation with key tumor immunity genes. (**A**) Comparison of AIMP2 expression with immune-related gene groups. (**B**) Correlation analysis between AIMP2 expression and tumor-related regulatory activities.

To investigate the relationship between AIMP2 expression and 33 tumor immune-related genes, we performed gene co-expression analysis in this study. The analyzed genes comprised MHC molecules, immune activators, immune suppressors, chemokines, and chemokine receptor proteins. Our results demonstrated that AIMP2 was significantly associated with almost all immune-related genes (Fig. **4A**). Moreover, AIMP2 showed significant correlations with common tumor-associated regulatory genes, such as TGF BETA SIGNALING, TNFA SIGNALING, hypoxia, pyroptosis, DNA repair, autophagy genes, and ferroptosis-related genes (Fig. **4B**).

### 3.4 Pan-cancer Expression and TMB and MSI

**Fig. (5):**
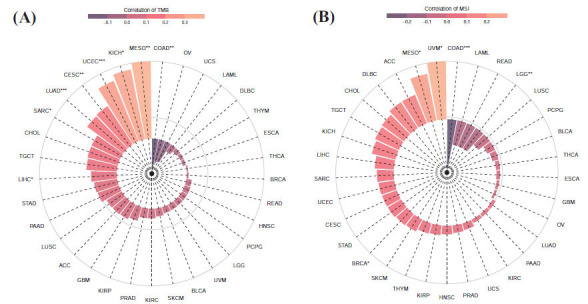
Correlation analysis between immune-related biomarkers and AIMP2 in different cancers. (**A**) AIMP2 was significantly correlated with TMB tumors in COAD, LIHC, SARC, LUAD, CESC, UCEC, KICH, and MESO. (**B**) AIMP2 expression was significantly correlated in COAD, LGG, BRCA, MESO, and UVM with MSI.

TMB and microsatellite instability (MSI) are emerging biomarkers linked to immunotherapy responses. This study investigated the association between AIMP2 expression and TMB and MSI across various cancers. Our findings revealed a significant correlation between AIMP2 expression levels and TMB in COAD, LIHC, SARC, LUAD, CESC, UCEC, KICH, and MESO tumors (Fig. **5A**). In relation to MSI, AIMP2 expression was significantly different in COAD, LGG, BRCA, MESO, and UVM (Fig. **5B**).

### 3.5 Pan-cancer Expression and Drug Sensitivity

**Fig. (6):**
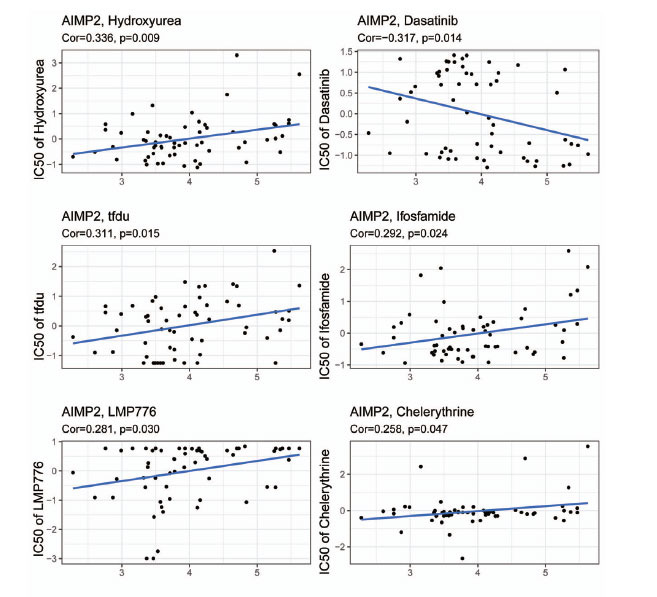
Correlation analysis between AIMP2 expression and anti-cancer drugs. Cor = correlation. Cor > 0 means the higher the gene expression, the greater the IC50 and the more resistance.

Surgery combined with chemotherapy is particularly effective in early-stage tumors. We examined the relationship between AIMP2 gene expression and the sensitivity to commonly used anti-cancer drugs using the CellMiner database. Our analysis indicated that high AIMP2 expression is associated with increased resistance to multiple anti-cancer drugs (Fig. **6**). Specifically, AIMP2 expression positively correlated with hydroxyurea, tfdu, ifosfamide, LMP776, and chelerythrine, while it negatively correlated with dasatinib.

### 3.6 Pan-cancer Expression and GSVA/GSEA

**Fig. (7):**
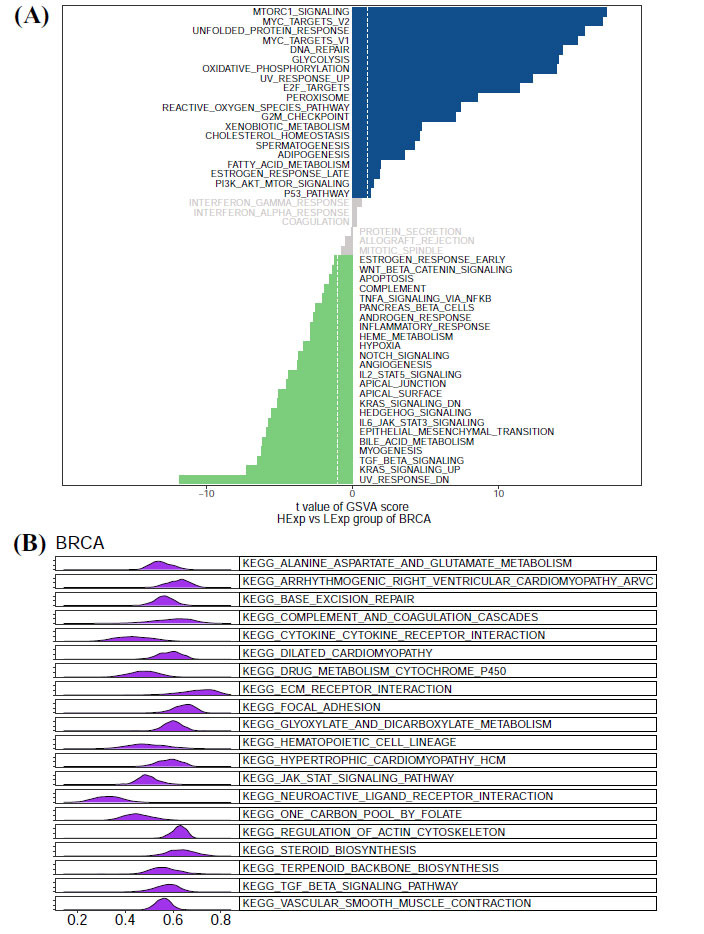
(**A**) Using the GSVA score, the correlation between AIMP2 and signaling pathways in BRCA was evaluated. (**B**) GSEA analysis of AIMP2 and enrichment pathways in BRCA.

To further investigate the molecular mechanisms of AIMP2 in pan-cancer, we employed GSVA to score all tumor samples and subsequently divided them into high and low-expression groups based on the median gene expression. Our results demonstrated that in breast cancer, high AIMP2 expression was predominantly found in MTORC1 signaling, MYC TARGETS_V2, UNFOLDED_ PROTEIN_RESPONSE, and other signaling pathways (Fig. **7A**). GSEA analysis of the relationship between AIMP2 and breast cancer is presented in Fig. (**7B**). Through KEGG analysis, high AIMP2 in BRCA is associated with ECM-receptor interaction, FOCAL adhesion, STEROID biosynthesis, and other enrichment pathways.

### 3.7 AIMP2 Risk and Independent Prognosis Analysis

**Fig. (8):**
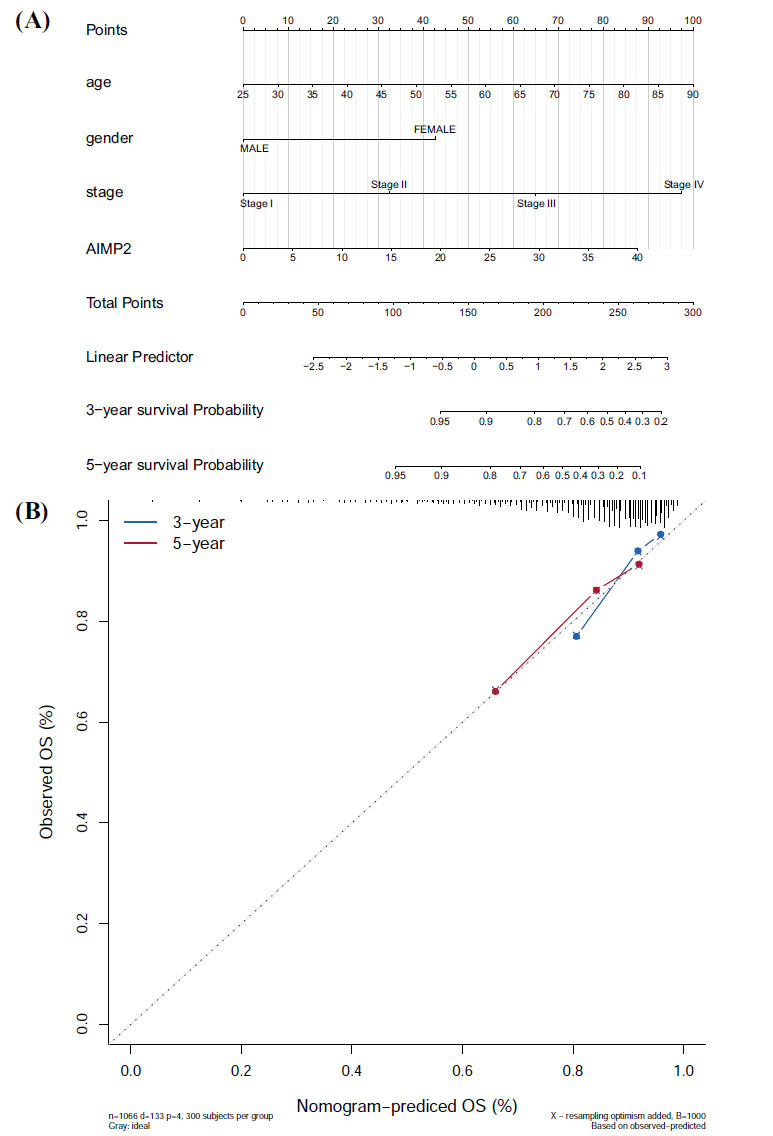
AIMP2 expression and independent prognosis analysis. (**A**) The nomogram prediction model shows that AIMP2 expression has good predictive performance. (**B**) The calibration curves show that the predicted OS has good agreement with the nomogram prediction model.

A nomogram prediction model was constructed based on AIMP2 expression and clinical features, and the results of the regression analysis were depicted as a nomogram. Logistic regression analysis revealed that in our BRCA samples, AIMP2 gene expression was a critical component of the model, contributing substantially to its predictive performance (Fig. **8A**). Moreover, the calibration curves demonstrated that the predicted 3- and 5-year OS rates were in strong agreement with the observed OS rates (Fig. **8B**).

### 3.8 WGCNA Network

**Fig. (9):**
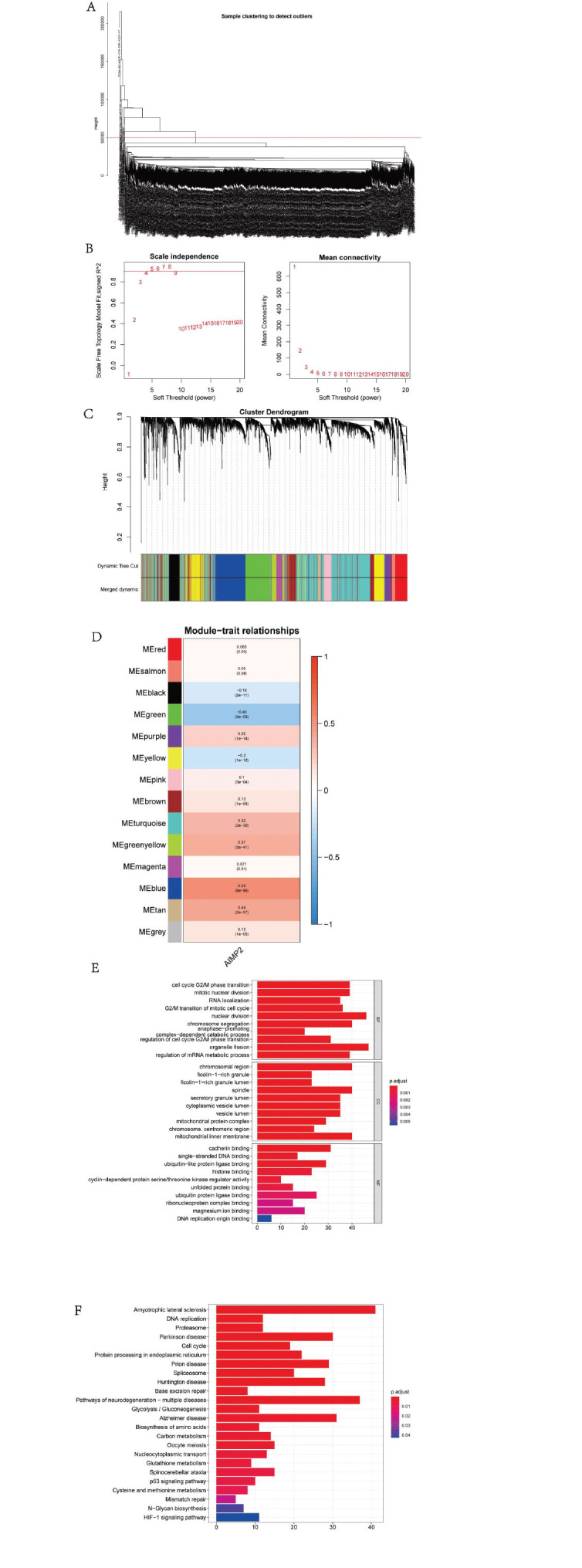
(**A, B**) The co-expression network related to AIMP2 in BRCA was explored based on the WGCNA network. The soft threshold β is determined by the function “sft$powerEstimate”. (**C**) TOM matrix was used to detect gene modules, and a total of 14 gene modules were detected. (**D**) The blue module had the highest correlation with AIMP2 (cor = 0.55, p = 8e−96). (**E**, **F**) GO and KEGG show the regions and pathways enriched for AIMP2.

To investigate the AIMP2-related co-expression network in breast cancer, we performed a WGCNA based on the BRCA dataset (Fig. **9A**). The soft threshold β was determined using the “sft$powerEstimate” function (Fig. **9B**), followed by gene module detection based on the TOM. This analysis identified 14 gene modules (Fig. **9C**). Subsequent analysis of module-trait relationships revealed that the blue module had the highest correlation with AIMP2 (cor = 0.55, p = 8e−96) (Fig. **9D**). GO analysis demonstrated significant enrichment in pathways, such as cell cycle G2/M phase transition, mitotic nuclear division, and RNA localization (Fig. **9E**). KEGG analysis indicated major enrichment in pathways, including amyotrophic lateral sclerosis, DNA replication, and proteasome (Fig. **9F**).

### 3.9 AIMP2 Knockdown Suppresses Proliferation, Migration, and Invasion in BRCA Cells

**Fig. (10):**
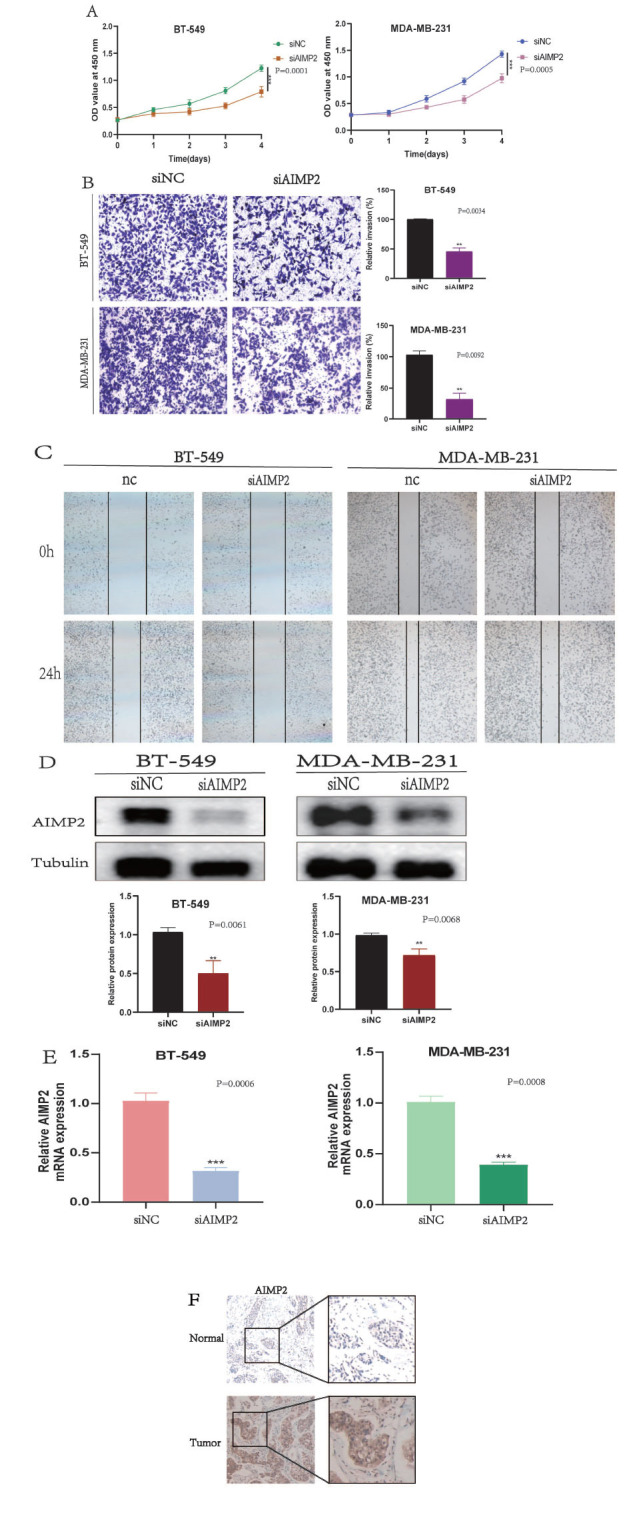
Cellular functions of AIMP2. (**A**) CCK-8 assay results show a decrease in the viability of BT-549 and MDA-MB-231 cells upon AIMP2 knockdown (***, *p* < 0.001). (**B**) Transwell assay results show a decrease in the invasion ability in BT-549 and MDA-MB-231 cells upon AIMP2 knockdown (**, *p* < 0.01). (**C**) Scratch assay showed that AIMP2 knockdown decreased the migration ability of BT-549 and MDA-MB-231 cells. (**D, E**) Western blot and real-time quantitative PCR detection show the knockdown efficiency of AIMP2 (**, *p* < 0.01; ***, *p* < 0.001). (**F**) AIMP2 expression in normal and tumor tissues.

Our CCK-8 assay demonstrated that AIMP2 knockdown significantly reduced the proliferation of BT-549 and MDA-MB-231 cells (Fig. **10A**). Transwell assay results revealed a decrease in invasion capacity following AIMP2 knockdown (Fig. **10B**). The wound healing assay indicated a reduced migration ability in the siAIMP2 group compared to the siNC group after 24 hours (Fig. **10C**). Cells were collected for qRT-PCR and western blot analysis after 48 hours of transfection, confirming successful transfection (Figs. **10D** and **E**) (**, *P* < 0.01; ***, *P* < 0.001). Immunohistochemistry revealed that AIMP2 was highly expressed in tumor tissue and exhibited low expression in normal tissue (Fig. **10F**).

## 4 DISCUSSION

Our study demonstrated that the AIMP2 gene is highly expressed in 24 types of cancer, with IHC analysis corroborating this trend at the protein level in BRCA. The results for lung cancer align with those of previous research [25]; however, Kim *et al.* reported that AIMP2 expression was reduced in gastric and colorectal cancer compared to their paired tissues, which contradicts our findings and indirectly confirms the complex mechanism of AIMP2 in different tumors [26]. In other tumors, there is a lack of research on AIMP2 expression. Our study establishes that aberrant AIMP2 expression occurs in numerous tumor types. Kaplan-Meier survival analysis using TCGA data revealed that elevated AIMP2 expression is associated with poor prognosis in ACC, BLCA, BRCA, HNSC, and UCEC. However, our investigation of the database revealed a limited number of studies focusing on these five tumor types.

Furthermore, we found that AIMP2 expression is associated with cancer stage in specific tumor types. A positive correlation between AIMP2 expression and tumor stage in ACC, BRCA, and LUAD was observed, which could inform immunotherapy selection for patients with different stages of these cancers. Additionally, we constructed a nomogram prediction model based on AIMP2 gene expression and clinical symptoms. Logistic regression analysis revealed that AIMP2 gene expression was significant in our BRCA samples, and the calibration curves displayed a strong agreement between the predicted 3- and 5-year OS and the observed OS. These results indicated that AIMP2 can serve as a prognostic biomarker for these three kinds of cancer. Moreover, our study examined the relationship between AIMP2 gene expression and common anti-tumor drugs using the CellMiner database, revealing that high AIMP2 expression is predicted to correlate with tolerance to multiple anti-tumor drugs. Notably, AIMP2 expression is positively associated with hydroxyurea, tfdu, ifosfamide, LMP776, and chelerythrine, and negatively correlated with dasatinib. These findings suggested that assessing AIMP2 expression levels may be crucial for evaluating patient conditions and selecting an appropriate treatment strategy.

TMB has emerged as a promising pan-cancer predictive biomarker [27] with the potential to guide immunotherapy in the era of precision medicine [28]. Previous studies have shown that TMB can enhance immunotherapy efficacy in non-small cell lung and colorectal cancers [29, 30] and predict prognosis following immunotherapy in pan-cancer patients [31]. Microsatellite instability (MSI) is another important biomarker for immune checkpoint inhibitors (ICI) [29, 32]. High-frequency MSI in colorectal cancer independently predicts clinical characteristics and prognosis [33]. Our study demonstrated that AIMP2 expression correlates with TMB in eight cancer types and with MSI in five cancer types. This suggests that AIMP2 expression levels may influence TMB and MSI in cancer, thereby affecting a patient's response to immune checkpoint suppression therapy and providing a novel reference for immunotherapy prognosis. Considering existing research and our findings, we hypothesize that tumors with high AIMP2 expression and elevated TMB and MSI levels may exhibit improved prognosis following ICI treatment in cancers where AIMP2 expression positively correlates with TMB.

Our results demonstrated that AIMP2 plays a crucial role in cancer immunity. Features of the tumor microenvironment (TME) serve as markers for assessing tumor cell responses to immunotherapy and influence clinical outcomes [34]. Tumor-infiltrating immune cells significantly impact tumor occurrence and development, either promoting or antagonizing these processes [35]. Our findings revealed a strong association between AIMP2 expression and immune infiltration. Specifically, we observed significant associations with macrophages M0 cells in 14 cancers, macrophages M2 cells in 10 cancers, and macrophages M1 cells in 8 cancers. We conducted further TME analysis in BRCA and discovered significant correlations between TMEscore, Antigen_processing_machinery, TMEscoreA, Mismatch_ Repair, Nucleotide_excision_repair, DNA_damage_response, DNA_replication, Base_excision_repair, Pan_F_TBRs, EMT1, EMT2, and TMEscoreB scores with BRCA. Additionally, we performed gene co-expression analysis to investigate the relationship between AIMP2 expression and 33 tumor immune-related genes, including MHC, immune activators, immune suppressors, chemokines, and chemokine receptor proteins. Our results demonstrated significant associations between AIMP2 and nearly all immune-related genes. Moreover, AIMP2 was found to be significantly correlated with common tumor-related regulatory genes, such as TGF BETA SIGNALING, TNFA SIGNALING, hypoxia, pyroptosis, DNA repair, autophagy genes, and ferroptosis-related genes.

To further investigate the molecular mechanism of the AIMP2 gene in BRCA, we conducted GSVA and GSEA analyses and constructed a WGCNA network. Our results revealed high AIMP2 expression primarily concentrated in MTORC1_SIGNALING, MYC_TARGETS_V2, UNFOLDED_PROTEIN_RESPONSE, and other signaling pathways. WGCNA network results indicated that genes in the module exhibiting the highest correlation with AIMP2 were mainly enriched in pathways, such as cell cycle G2/M phase transition, mitotic nuclear division, and RNA localization. KEGG results demonstrated that genes were primarily enriched in pathways, such as amyotrophic lateral sclerosis, DNA replication, and proteasome. *In vitro* cell experiments indicated that AIMP2 expression in tumor tissues was higher than in normal tissues, and inhibiting AIMP2 expression could affect the biological behavior of breast cancer cells.

## CONCLUSION

In conclusion, our first pan-cancer analysis of AIMP2 revealed differential expression between tumor and normal tissues. Our findings suggested that AIMP2 may serve as an independent prognostic factor for various tumors, particularly BRCA. The specific role of AIMP2 in each cancer warrants further investigation, as its expression levels may result in different prognostic outcomes. Furthermore, AIMP2 expression was associated with TMB, MSI, and immune cell infiltration across multiple cancer types, with its impact on tumor immunity varying among tumor types. These findings may help to clarify the role of AIMP2 in tumorigenesis and development, potentially informing more precise and personalized immunotherapy approaches in the future.

## Data Availability

The datasets generated during this study are available from the corresponding author upon reasonable request.

## LIST OF ABBREVIATIONS

BRCA: Breast Cancer
CCK-8: Cell Counting kit 8
CCLE: Cancer Cell Lines Encyclopedia
GSEA: Gene Set Enrichment Analysis
GSVA: Gene Set Variation Analysis
GTEx: Genotype-tissue Expression
HRs: Hazard Ratios
ICI: Immune Checkpoint Inhibitors
MHC: Major Histocompatibility Complex
MSI: Microsatellite Instability
NCI: National Cancer Institute's
OS: Overall Survival
SNPs: Single Nucleotide Polymorphisms
TCGA: The Cancer Genome Atlas
TISIDB: Tumor-immune System Interactions Data- base
TMB: Tumor Mutation Burden
TME: The Tumor Microenvironment
TOM: Topological Overlap Matrix
