# Supplementary material for: Pan-cancer Analysis Identifies AIMP2 as a Potential Biomarker for Breast Cancer
Source: Curr Genomics. 2023 Dec 20;24(5):307–29. doi: 10.2174/0113892029255941231014142050 (PMC10790333; doi:10.2174/0113892029255941231014142050)
Supplement: Supplementary file 1 [file CG-24-307_SD1.pdf]

Supplementary Material

Pan-cancer Analysis Identifies AIMP2 as a Potential Biomarker for Breast Cancer

Jie Qiu[1,#], Tao Zhou[2,#], Danhong Wang[3], Weimin Hong[2], Da Qian[4,*], Xuli Meng[2,*] and Xiaozhen Liu[2,*]

[1]Department of Breast and Thyroid Surgery, Shaoxing People's Hospital, Shaoxing 312000, Zhejiang, China; [2]General Surgery, Cancer Center, Department of Breast Surgery, Zhejiang Provincial People's Hospital, Hangzhou Medical College, Hangzhou 310000, Zhejiang, China; [3]College of Pharmacy, Zhejiang University of Technology, Hangzhou 310014, Zhejiang, China; [4]Department of Burn and Plastic Surgery-Hand Surgery, Changshu Hospital Affiliated to Soochow University, Changshu No.1 People's Hospital, Changshu 215500, Jiangsu Province, China

ACC（N=0, T=79）, BLCA（N=19, T=414）, BRCA（N=113, T=1109）,CESC（N=3, T=306), CHOL (N=9, T=36),COAD (N=41, T=480),DLBC(N=48, T=48),ESCA(N=11,T=162),GBM(N=5,T=169), HNSC（N=44, T=502）, KICH（N=24,T=65）, KIRC（N=72, T=539）, KIRP（N=32,T=289）LAML（N=0,T=151, LGG（N=0,T=529）,LIHC (N=50, T=374),LUAD (N=59, T=535),LUSC (N=49,T=502),MESO (N=0, T=86),OV (N=0, T=379),PAAD (N=4, T=178),PCPG (N=3, T=183),PRAD (N=52,T=499),READ(N=10 T=167),SARC(N=2, T=263),SKCM (N=1, T=471),STAD(N=32,T=375),TGCT (N=0, T= 156),THCA (N=58 , T=510),THYM (N=2, T=119),UCEC (N=35, T=552), UCS (N=0,T=56), UVM (N=0, T=80)

Supplementary material S1.

SymbolACC(N=128,T=79),symbolBLCA(N=28,T=414),symbolBRCA(N=292,T=1109),symbolCESC(N= 13,T=306), symbolCHOL(N=9,T=36),symbolCOAD(N=349,T=480),symbolDLBC(N=0,T=48),symbolES CA(N=664,T=162), symbolGBM(N=1157,T=169),symbolHNSC(N=44,T=502),symbolKICH(N=52,T=6 5),symbolKIRC(N=72,T=539), symbolKIRP(N=32,T=289),symbolLAML(N=337,T=151),symbolLGG(N=1152,T=529),symbolLIHC(N=160,T=374),symbolLUAD (N=59,T=535),symbolLUSC(N=49,T=502),symbolMESO(N=0,T=86),symbolOV(N=88,T=379),symbolPAAD(N=171,T=17 8),symbolPCPG(N=3,T=183),symbolPRAD(N=152,T=499),symbolREAD(N=10,T=167),symbolSARC(N=2,T=263),symbol SKCM(N=557,T=471),symbolSTAD(N=206,T=375),symbolTGCT(N=165,T=156),symbolTHCA(N=337,T510),symbolTHY M(N=2,T=119),symbolUCEC(N=35,T=552),symbolUCS(N=78,T=56),symbolUVM(N=0,T=80)

Supplementary material S2.

Thyroid Cancer(17),Liposarcoma(10),Engineered(13),Cervical Cancer(17),Leukemia(100),Fibroblast(39),Myeloma(30), Head and Neck Cancer(54),Gallbladder Cancer(6),Breast Cancer(61),Bile Duct Cancer(35),Lymphoma(82),Bone Cancer(32), Prostate Cancer(11),Kidney Cancer(37),Rhabdoid(17),Bladder Cancer(36),Eye Cancer(9),Ovarian Cancer(64), Sarcoma(34), Pancreatic Cancer(52),Neuroblastoma(27),Skin Cancer(83),Endometrial/Uterine Cancer(39),Colon/ Colorectal Cancer(70), Esophageal Cancer(32),Liver Cancer(24),Lung Cancer (204),Gastric Cancer(40),Brain Cancer(83).

Supplementary material S3.
